# Rice Husk Silica Liquid Protects Pancreatic β Cells from Streptozotocin-Induced Oxidative Damage

**DOI:** 10.3390/antiox10071080

**Published:** 2021-07-05

**Authors:** Hsin-Yuan Chen, Yi-Fen Chiang, Kai-Lee Wang, Tsui-Chin Huang, Mohamed Ali, Tzong-Ming Shieh, Hsin-Yi Chang, Yong-Han Hong, Shih-Min Hsia

**Affiliations:** 1 School of Nutrition and Health Sciences, College of Nutrition, Taipei Medical University, Taipei 11031, Taiwan; d507104002@tmu.edu.tw (H.-Y.C.); da07108002@tmu.edu.tw (Y.-F.C.); 2 Department of Nutrition, I-Shou University, Kaohsiung 84001, Taiwan; 3 Department of Nursing, Ching Kuo Institute of Management and Health, Keelung 20301, Taiwan; kellywang@tmu.edu.tw; 4 Graduate Institute of Cancer Biology and Drug Discovery, College of Medical Science and Technology, Taipei Medical University, Taipei 11031, Taiwan; tsuichin@tmu.edu.tw; 5 Clinical Pharmacy Department, Faculty of Pharmacy, Ain Shams University, Cairo 11566, Egypt; mohamed.aboouf@pharma.asu.edu.eg; 6 School of Dentistry, College of Dentistry, China Medical University, Taichung 40402, Taiwan; tmshieh@mail.cmu.edu.tw; 7 Graduate Institute of Metabolism and Obesity Sciences, College of Nutrition, Taipei Medical University, Taipei 11031, Taiwan; hsinyi.chang@tmu.edu.tw; 8 School of Food and Safety, Taipei Medical University, Taipei 11031, Taiwan; 9 Nutrition Research Center, Taipei Medical University Hospital, Taipei 11031, Taiwan

**Keywords:** rice husk silica liquid, streptozotocin, pancreatic β cell, reactive oxygen species, autophagy, apoptosis

## Abstract

Type 2 diabetes mellitus is a complex multifactorial disease characterized by insulin resistance and dysfunction of pancreatic β-cells. Rice husk silica liquid (RHSL) is derived from rice husks and has not been explored in diabetes mellitus until now. Previous studies showed that rice husk is enriched with silica, and its silica nanoparticles are higher more biocompatible. To investigate the potential protective role of RHSL on pancreatic β cells, we utilized RIN-m5F pancreatic β cells and explored RHSL effect after streptozotocin (STZ)-stimulation. The recovery effects of RHSL were evaluated using flow cytometry, Western blotting, and immunofluorescence analysis. Results of our study showed that RHSL reversed the cell viability, insulin secretion, reactive oxygen species (ROS) production, and the change of mitochondria membrane potential (ΔΨm) in STZ-treated RIN-m5F cells. Moreover, the expression of phospho-receptor-interacting protein 3 (p-RIP3) and cleaved-poly (ADP-ribose) polymerase (PARP), phospho-mammalian target of rapamycin (p-mTOR), and sequestosome-1 (p62/SQSTM1) were significantly decreased, while the transition of light chain (LC)3-I to LC3-II was markedly increased after RHSL treatment in STZ-induced RIN-m5F cells. Interestingly, using autophagy inhibitors such as 3-methyladenine (3-MA) and chloroquine (CQ) both showed an increase in cleaved-PARP protein level, indicating apoptosis induction. Taken together, this study demonstrated that RHSL induced autophagy and alleviated STZ-induced ROS-mediated apoptosis in RIN-m5F cells.

## 1. Introduction

Type 2 diabetes mellitus (T2DM) is a chronic metabolic disorder that has become a global concern [1]. The major pathogenesis involves the loss or destruction of pancreatic β cells, which in turn induces the state of imbalance between insulin secretion and blood sugar control in the body [2]. Streptozotocin (STZ) is a kind of glucosamine-nitrosourea compound and widely used as a broad-spectrum antibiotic and cytotoxic chemical [3]. Due to its structural similarity to 2-deoxy-D-glucose, it can enter pancreatic β cells through the GLUT2 glucose transporter and efficiently accumulates intracellularly. Intracellular accumulation induces DNA alkylation and exposes the cells to reactive oxygen species (ROS) and nitric oxide (NO) with subsequent damage, ultimately provoking diabetogenic action [4]. Diabetes induced by STZ is the most similar to the structural, functional and biochemical changes observed in human diabetes, so it is often used to simulate diabetes models in animal experiments [4,5] as well as to verify the mechanism of pancreatic β-cells in in vitro experiments [6], which involve mitochondrial dysfunction, ROS production, necrosis, and apoptosis.

Silicon (Si) is known as an essential component of collagen and glycosaminoglycan formed in bones and cartilage [7,8]. Several studies also demonstrated that dietary Si intake has benefits on bone mineral density [9,10]. In addition, Si showed an anti-diabetic effect by lowering blood glucose level, improving the tolerance to insulin and reducing the risk of glomerulopathy [11]. Previous studies have demonstrated rice husk is enriched with silica, and its silica nanoparticles are more biocompatible [12], thus expecting more applications in the food industry. However, food inspection and functional analysis of rice husk silica (RHS) remain insufficient. Furthermore, the potential anti-diabetic role of rice husk silica liquid (RHSL) on pancreatic β cell has not been explored by any research so far. Herein, we designed the present study to further investigate the mechanisms by which RHSL can protect against T2DM, aiming to shed new light on T2DM prevention by plant-derived silica.

## 2. Materials and Methods

### 2.1. Reagent Preparation

The detailed process of rice husk preparation is shown in previous studies [13]. Briefly, the production of rice husk silica (RHS) involves smoldering rice husk samples (PIN FU FA Corporation, Yunling, Taiwan) at a temperature of 300 °C for 2 h in a low-oxygen environment (oxygen concentration less than 5%), and then firing them at a temperature of 800 °C for 2 h in the atmosphere. Finally, an aqueous RHSL (rice husk silica liquid) is obtained through an alkali solution processing process. The main element of the sample is silicon (Si). We identified the RHSL through Inductively Coupled Plasma Mass Spectrometry (ICP-MS, Thermo-Element XR, Thermo Fisher Scientific, Waltham, MA, USA), and the Si content was measured to be 9800 ± 150 µg/mL. Based on this, the standardized component was used as the original concentration and dilute with deionized water into an effective dose in subsequent experiments.

Streptozotocin (STZ, C_8_H_15_N_3_O_7_; MW: 265.2; CAS number: 18883-66-4; purity ≥ 95%) was obtained from Cayman Chemical (Ann Arbor, MI, USA), which is a white to light yellow crystalline solid. STZ was reconstituted in sodium-citrate buffer (pH 4.5) containing 0.1 M Na-Citrate (Sigma-Aldrich, St. Louis, MO, USA) and 0.1 M Citric acid (Sigma-Aldrich, St. Louis, MO, USA) at 500 mM and stored at −20 °C until use. The carrier solvent (0.4% sodium-citrate buffer) was added to the control group.

### 2.2. Cell Culture

RINm5F Insulinoma cells (CRL-11605; American Type Culture Collection, ATCC, Manassas, VA, USA) were cultured in RPMI 1640 (ATCC, Manassas, VA, USA) supplemented with 10% fetal bovine serum (FBS; CORNING, Manassas, VA, USA) at 37 °C in a humidified atmosphere containing 5% CO_2_. Cell culture medium was collected and checked for mycoplasma using the EZ-PCR-Mycoplasma Test Kit (Biological Industries, Cromwell, CT, USA). RINm5F cells were divided into six groups: (a) control group, cells were incubated 0.4% sodium-citrate buffer; (b) RHSL 200× group, cells were incubated with RHSL diluted 200 times; (c) RHSL 100× group, cells were incubated with RHSL diluted 100 times; (d) STZ stimulated group, cells were incubated with 2 mM STZ; (e–f) STZ combined with RHSL treatment groups, cells were co-treated with RHSL (200× and 100×) and/or 2 mM STZ.

### 2.3. Cell Viability

The influence of STZ and RHSL on RIN-m5F cell viability was analyzed by the (3-(4,5-Dimethylthiazol-2-yl)-2,5-diphenyltetrazolium bromide) (MTT) assay (Sigma-Aldrich, St. Louis, MO, USA). Cells were seeded in 96-well plates (1 × 10^4^ cells per well) and administrated as follows: STZ group, treated with various dosages (2 mM and 4 mM) of STZ or sodium-citrate buffer (0.8%) alone as vehicle control; RHSL group, treated with various dosages (400×, 200×, and 100×) of RHSL or sterile water alone as vehicle control. After 24 and 48 h, the medium was replaced by 100 μL of medium containing 1% serum with 0.5 mg/mL MTT. Following a 3-h incubation at 37 °C, formazan crystals were solubilized with 100 μL of DMSO. The absorbance levels for each sample at 570 nm with reference wavelength of 630 nm were measured using a microplate reader (Bio-Tek, Winooski, VT, USA). The experimental data is presented in triplicates.

### 2.4. Insulin Secretion Assay by ELISA Kit

RIN-m5F cells were seeded in 24-well plates (5 × 10^5^ cells per well) and treated with different doses of RHSL (200× and 100×) and STZ 2 mM for 24 h. At the end of this incubation period, culture medium was collected and the content of insulin was measured by a commercial enzyme-linked immunosorbent assay (ELISA) kit (Mercodia; 10-1250 [rat], Uppsala, Sweden) according to the manufacturer’s instructions. The absorbance levels for each sample at 450 nm were measured using a microplate reader (Bio-Tek, Winooski, VT, USA). The experimental data is presented in triplicates.

### 2.5. Silicon Level Determination

RIN-m5F cells were seeded in a 10 cm^2^ dish at a density of 1 × 10^6^ and treated with different doses of RHSL (200× and 100×) and STZ 2 mM for 24 h. At the end of this incubation period, supernatant and cell pellets were collected separately, and the Si content is determined by the Center of Aquatic Product Inspection and Certification (National Kaohsiung University of Science and Technology). Briefly, the silicon content is determined by Thermo Scientific iCAP PRO Inductively Coupled Plasma Optical Emission Spectroscopy (ICP-OES, Thermo Fisher Scientific, Waltham, MA, USA) equipped with a cyclonic spray chamber. Analysis was performed in axial mode depending on the element and matrix. Vortex all samples thoroughly to provide a homogeneous matrix for digestion. The sample volume of 0.1 mL of each was digested with 1.0 mL H_2_O_2_ and quantified to 5.0 mL with 1% (*v*/*v*) HNO_3_. Calibration standards for ICP-OES analysis were prepared by dilution of 1000 mg/L Si standards.

### 2.6. Measurement of Reactive Oxygen Species (ROS) Production

To quantitatively assess intracellular ROS in response to RHSL in STZ-induced RIN-m5F cells, the indicator 6-carboxy-2’,7’-dichlorodihydrofluorescein diacetate (carboxy-H_2_DCFDA; Invitrogin™, Thermo Fisher Scientific, Waltham, MA, USA) was used. Briefly, RIN-m5F cells were seeded in 6-well plate (1 × 10^5^ cells per well), induced by STZ and exposed to RHSL. Then, RIN-m5F cells were harvested and washed twice with ice-cold PBS, and then incubated with 20 µM carboxy-H_2_DCFDA in serum-free medium at 37  °C in dark for 45 min. Subsequently, the stained cells are washed once to remove the residual dye and kept on ice for later use. For H_2_O_2_ positive control treatment, the medium was refreshed with 1% serum containing 1 mM H_2_O_2_ and incubated for 30 min. ROS levels were detected at the FL-1 channel using Attune™ NxT Flow Cytometer (Thermo Fisher Scientific, Waltham, MA, USA). The experimental data is presented in triplicates.

### 2.7. Mitochondria Membrane Potential (ΔΨm) Assay

To assess the change of ΔΨm in response to RHSL in STZ-induced RIN-m5F cells, fluorescence probes 5, 5, 6, 6′-tetrachloro-1, 1′, 3, 3′ tetraethylbenzimidazoyl-carbocyanine iodide (JC-1; Invitrogin™, Thermo Fisher Scientific, Waltham, MA, USA) were used. Briefly, RIN-m5F cells were seeded in 6-well plate (1 × 10^5^ cells per well), induced by STZ and exposed to RHSL. Subsequently, RIN-m5F cells were harvested and washed twice with ice-cold PBS before stained with JC-1 fluorescence probes at 37 °C, 5% CO_2_, for 15 min. The intensity of green fluorescent (monomeric) and red fluorescent (aggregated) was detected at FL-1 and FL-2 channels, respectively, and analyzed by Attune™ NxT Flow Cytometer (Thermo Fisher Scientific, Waltham, MA, USA). The experimental data is presented in triplicates.

### 2.8. Western Blotting Analysis

RIN-m5F cells were seeded into 10 cm^2^ dish at a density of 1 × 10^6^, induced by STZ, 3-methyladenine (3-MA), chloroquine (CQ)and exposed to RHSL. Cell lysates were lysed with 100 μL of lysis buffer (mixed by protease and phosphatase inhibitor cocktail (Roche, Basel, Switzerland)). Samples containing 20 μg of proteins were subjected to SDS-PAGE gels and transferred to a PVDF membrane (0.45 µm or 0.22 µm, for 100 min at 100 V). The membranes were blocked with 5% bovine serum albumin (BSA; BioShop, Burlington, ON, Canada) for 1 h, and then probed with primary antibodies at 4 °C overnight, including: anti-poly (ADP-ribose) polymerase (PARP), anti-light chain 3 (LC3B), anti-sequestosome-1 (p62/SQSTM1), and anti-phospho-mammalian target of rapamycin (p-mTOR) which were purchased from Cell Signal Technology (Beverley, MA, USA); anti-phospho-receptor-interacting protein (p-RIP3) and anti-RIP3 were purchased from Affinity Biosciences (Melbourne, Victoria, Australia); anti-horseradish peroxidase (HRP)-conjugated glyceraldehyde 3-phosphate dehydrogenase (GAPDH) was purchased from Proteintech (Rosemont, IL, USA). Subsequently, membranes were incubated with the corresponding goat anti-rabbit/mouse antibody IgG (Abcam, Cambridge, UK) for 1 h. The chemiluminescence imaging was detected by the eBlot Touch Imagertm (eBlot Photoelectric Technology, Shanghai, China) after reacted with electrochemiluminescence (ECL; Thermo Fisher Scientific, Waltham, MA, USA). Densitometric estimations were quantified using the Image J software (National Institutes of Health, NIH, Bethesda, MD, USA).

### 2.9. Immunofluorescence Assay

To assess the formation of autophagosomes following RHSL treatment in STZ-treated RIN-m5F cells, immunofluorescence staining was used. RIN-m5F were seeded into 6-well plate (1 × 10^4^ cells per well) containing the sterile glass coverslips and treated with different doses of RHSL (200× and 100×) and STZ 2 mM for 24 h. Coverslips with cells were fixed with 4% paraformaldehyde for 10 min and then permeabilized with 0.1% Triton X-100 (Sigma-Aldrich, St. Louis, MO, USA)/PBS for 15 min. After blocking with 5% BSA (BioShop, Burlington, ON, Canada) buffer in 1× TBST for 30 min, coverslips with cells were incubated with primary antibody, anti-LC3B and anti-p62/SQSTM1, overnight at 4 °C. Coverslips with cells were subsequently incubated at 25 °C for 45 min with a secondary antibody (Alexa Fluor^®^ 488 dye: Life Technologies, Gaithersburg, MD, USA). ProLong^®^ Gold Antifade Mountant (Thermo Fisher Scientific, Waltham, MA, USA) was added to the cells and coverslips were mounted on glass slides. Fluorescent imaging was performed under an inverted fluorescence microscope (Leica, Wetzlar, Germany).

### 2.10. Statistical Analysis

The data were expressed as the mean ± standard deviation (SD) and analyzed using the SigmaPlot, version 12.5 (SoftHome, Taipei, Taiwan, China). Statistical significance was performed by two-tailed Student’s *t* tests (two groups). The difference between two means was considered statistically significant when *p* < 0.05 and highly significant when *p* < 0.001.

## 3. Results

### 3.1. The Effect of RHSL and STZ on RIN-m5F Cells Viability

**Figure 1:**
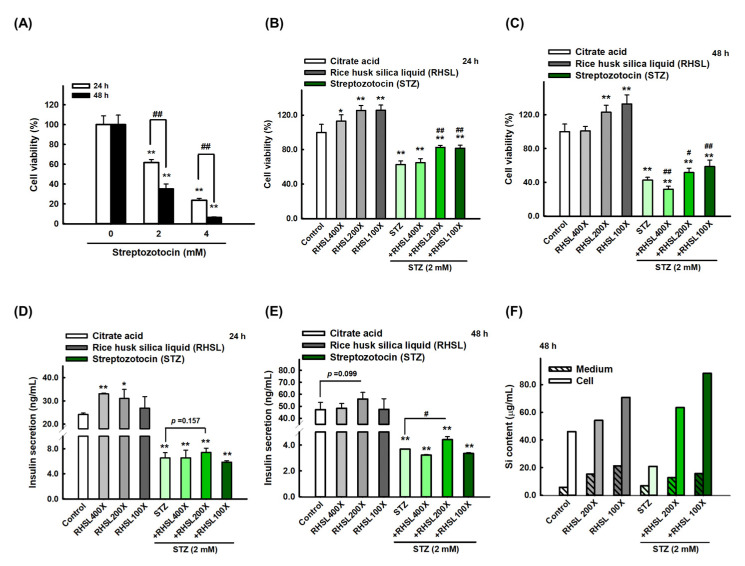
Rice husk silica liquid (RHSL) reverses streptozotocin (STZ)- induced cell viability and insulin secretion in RIN-m5F cells. (**A**) Screen the dosage of STZ (2 and 4 mM) for cell viability at 24 and 48 h. (**B**,**C**) The effect of RHSL and STZ treatment on cell viability for 24 and 48 h. (**D**,**E**) The amount of insulin (ng/mL) at 2 mM of STZ when treated with different dilutions of RHSL (400×, 200×, and 100×). (**F**) The level of silicon in cell pellet or culture medium at 2 mM of STZ when treated with different dilutions of RHSL (400×, 200×, and 100×). * *p* < 0.05; ** *p* < 0.001 compare with each control group (citrate acid). # *p* < 0.05; ## *p* < 0.001 compare with STZ group.

To investigate the effects of STZ on RIN-m5F cells, cell viability was quantified after exposing the RIN-m5F cell to toxin STZ. Our data indicated that STZ caused cytotoxicity in a dose and time-dependent manners (Figure 1A, *p* < 0.001). Briefly, STZ at 2 mM for 24 h markedly reduced the viability of RIN-m5F cells to ~60% (Figure 1A, *p* < 0.001). Based on these results, all subsequent experiments in this study were performed using 2 mM STZ as the induction model. On the other hand, we used different doses of RHSL from 400-fold dilution to 100-fold dilution and observed that RHSL treatment can enhance cell viability, especially at 200-fold and 100-fold dilution, which both significantly increased cell viability compared to the control group for 24 and 48 h in a dose-dependent manner (Figure 1B,C, *p* < 0.001).

Considering the results of individual used doses, we exposed RIN-m5F cells to the combination treatment and found that adding RHSL caused a significant recovery in cell survival in STZ-treated cells. Briefly, STZ at 2 mM markedly reduced the viability of RIN-m5F cells to ~60% for 24 h (Figure 1B, *p* < 0.001), and to ~40% for 48 h (Figure 1C, *p* < 0.001), while the cell viability was significantly recovered after co-treatment with RHSL at 200-fold and 100-fold dilution (Figure 1B,C, *p* < 0.001). Therefore, these results suggest that RHSL possesses the potential ability to restore the damage resulted from STZ treatment.

### 3.2. STZ Inhibits Insulin Secretion and Silicon Content in RIN-m5F Cells Which Is Restored by RHSL Co-Treatment

Next, we measured the level of insulin secretion to explore the therapeutic efficacy of RHSL in STZ-treated RIN-m5F cells. Insulin secretion in the culture medium was significantly reduced after STZ treatment (Figure 1D,E, *p* < 0.001), while the 200-fold dilution of RHSL significantly restored the level of insulin after STZ treatment (Figure 1E, *p* < 0.05).

In the other hand, we accurately quantified the concentration of silicon (Si) in RIN-m5F cells and suspension after RHSL and STZ co-treatment. As expected, the RHSL group increased the Si content in the cells with or without STZ treatment (Figure 1F). Similar results were found in the suspension, that the RHSL group increased the Si content with or without STZ treatment (Figure 1F). Interestingly, compared to the control group, the treatment with STZ alone reduced the Si content in the cells (Figure 1F). Collectively, RHSL is shown to possess the potential ability to restore insulin secretion after STZ stimulation.

### 3.3. RHSL Protects RIN-m5F Cells Against STZ-induced ROS Production and Dysfunction of Mitochondria

**Figure 2:**
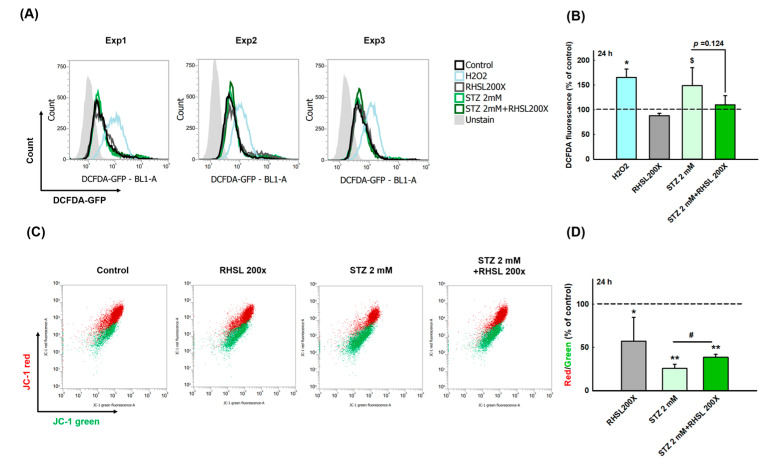
RHSL protects RIN-m5F cells against STZ-induced oxidant damage. Following treatment with RHSL in the absence or presence of STZ (2 mM) for 24 h. (**A**) The image of DCFDA fluorescence showed typical results of three independent experiments. (**B**) The DCFDA fluorescence intensity ratio represents the level of ROS production in RIN-m5F cells, and the dotted line represents the control group. (**C**) Distribution dot plot of mitochondrial depolarization stained by JC-1 (**D**) The red (~590 nm)/green (~529 nm) fluorescence intensity ratio represents depolarization of the mitochondrial membrane in RIN-m5F cells, and the dotted line represents the control group. $ *p* = 0.066; * *p* < 0.05; ** *p* < 0.001 compare with control group (citrate acid); # *p* < 0.05 compare with STZ group.

To explore whether RHSL can suppress the cell damage provoked by ROS in STZ-treated RIN-m5F cells, a cellular ROS assay was performed (Figure 2A). As a positive control, H_2_O_2_ accelerating the process of beta cell destruction through significantly increasing the ROS production (Figure 2B, *p* < 0.05). Even though the peak value was too large and there was no significant difference between the two groups, we still observe that STZ increases the ROS production in RIN-m5F cells (Figure 2B, *p* = 0.066), while RHSL slightly suppresses the ROS production caused by STZ (Figure 2B, *p* = 0.124).

Next, to investigate the changes of mitochondria membrane potential (ΔΨm) in STZ-treated RIN-m5F cells after RHSL intervention, a JC-1 assay was utilized (Figure 2C). We observed that the red/green ratio significantly decreased after STZ stimulation (Figure 2D, *p* < 0.001), while RHSL significantly restored the red/green ratio in STZ-treated RIN-m5F cells (Figure 2D, *p* <0.05). Therefore, these results have clearly indicated that RHSL possesses the potential ability to abrogate stress resulted from STZ stimulation.

### 3.4. RHSL Induces Autophagy and Attenuates Apoptosis in STZ-Induced RIN-m5F Cells

**Figure 3:**
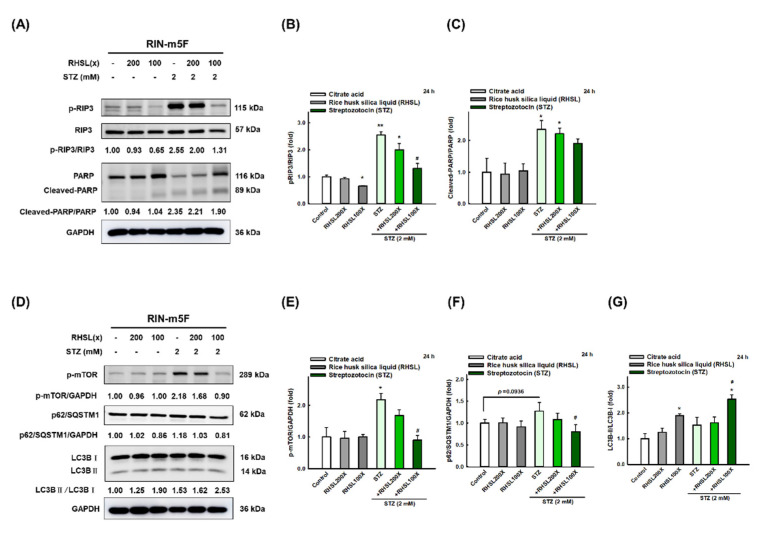
RHSL reverses STZ-induced cell death in RIN-m5F cells. Following treatment with RHSL in the absence or presence of (**A**,**D**) STZ (2 mM) for 24 h. Representative immunoblot for quantification of p-RIP3 (**B**), cleaved-PARP (**C**), p-mTOR (**E**), p62/SQSTM1 (**F**), and LC3B (**G**) expression in RIN-m5F. The optical densities of bands in each lane were normalized to GAPDH (loading control) optical density from the same gel. * *p* < 0.05; ** *p* < 0.001 compare with each control group (citrate acid). # *p* < 0.05 compare with STZ group.

To investigate which death pathways will be affected by RHSL under STZ stress, the markers of apoptosis, necroptosis, and autophagy were explored. We found that STZ induced cytotoxic effects in RIN-m5F cells through significantly enhancing the expression of cleaved-PARP (Figure 3A,C, *p* < 0.05; apoptosis-related marker) and p-RIP3 (Figure 3A,B, *p* < 0.001; necroptosis-related marker) protein at 24 h. Meanwhile, STZ inhibited autophagy via increasing the expression level of mTOR protein (Figure 3D,E, *p* < 0.05) and p62/SQSTM1 (Figure 3D,F, *p* = 0.0936), while no difference was found in the expression of LC3-II protein (Figure 3D,G). Interestingly, RHSL reversed the level of p-RIP3 (Figure 3A,B, *p* < 0.05) and cleaved-PARP (Figure 3A,C, *p* = 0.142) in STZ-treated RIN-m5F cells. Moreover, RHSL significantly promoted autophagy through decreasing p-mTOR (Figure 3A,C, *p* < 0.05) and p62/SQSTM1 (Figure 3D,F, *p* < 0.05) protein expression while increasing the conversion of LC3-I to LC3-II (Figure 3D,G, *p* < 0.05) in STZ-treated RIN-m5F cells. These results suggest that RHSL-induced autophagy may attenuate apoptosis.

### 3.5. RHSL Induces Autophagy in STZ-Induced RIN-m5F Cells

**Figure 4:**
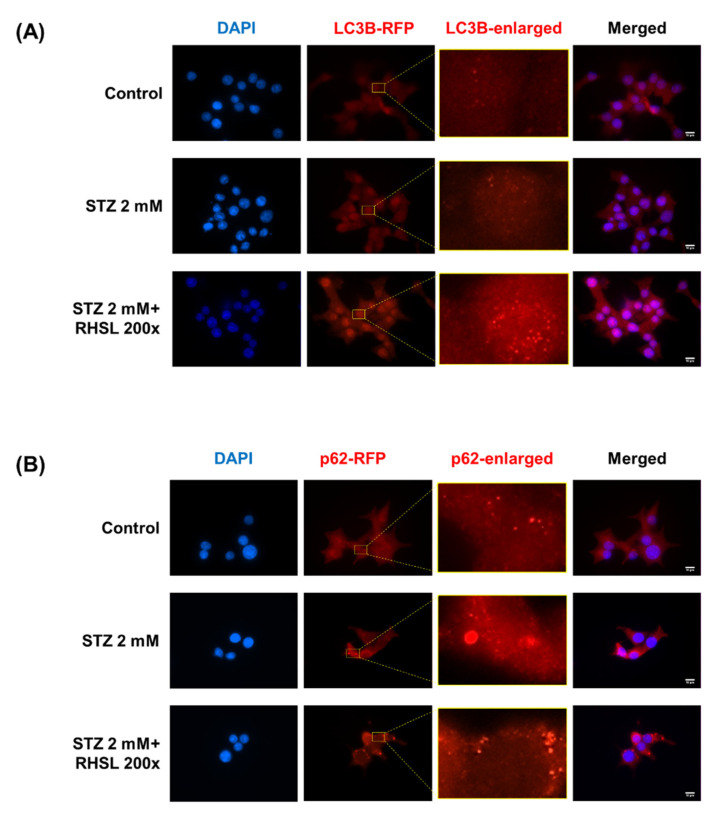
Effect of RHSL on STZ-induced autophagic disturbance in RIN-m5F cells. Representative microscopic images of LC3B (**A**) and p62/SQSTM1 (**B**) -immunostained RIN-m5F after STZ and RHSL treatment. Indicated rectangular areas magnified for clearer visualization of LC3 or p62/SQSTM1 puncta. Nuclear counterstaining was performed with 4’,6-Diamidino-2-phenylindole (DAPI). Calibration bar = 10 µm.

Next, an immunofluorescence assay was used to verify the changes in the expression of autophagy-related proteins in RIN-m5F cells. Typical characteristics of LC3 and p62/SQSTM1 are the appearance of cytosolic puncta, indicating the formation of autophagosomes [14]. There is no influence on the LC3B-staining group after STZ treatment, while RHSL increased the intensity of LC3 puncta (Figure 4A) in STZ-treated RIN-m5F cells. In the other hand, the results showed an increase in the strength of puncta in the p62-staining group after STZ treatment, indicating STZ induced p62 accumulation, while RHSL slightly reversed the intensity of puncta of p62/SQSTM1 (Figure 4B) in STZ-treated RIN-m5F cells. Collectively, these data demonstrated that RHSL attenuated STZ-induced autophagic disturbance and further stimulated autophagy.

### 3.6. RHSL Protects RIN-m5F Cells from Apoptosis through Autophagy Induction

**Figure 5:**
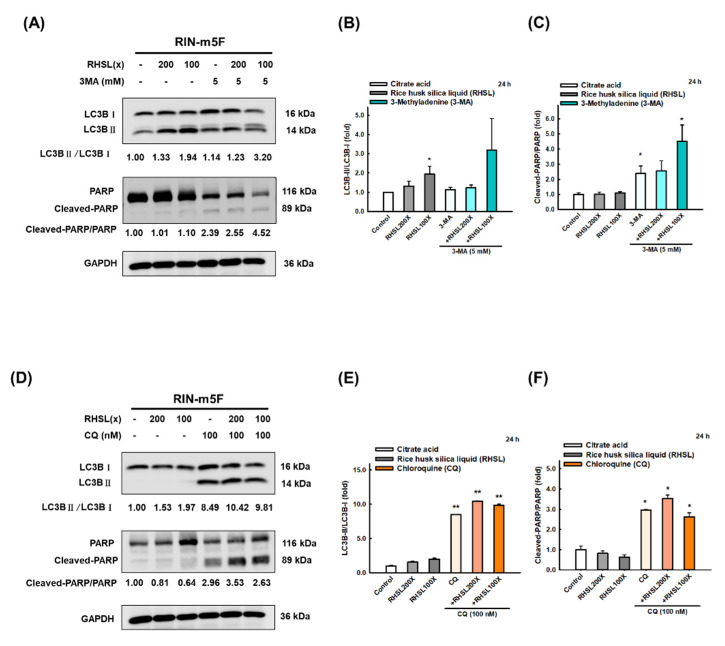
RHSL protects RIN-m5F cells from apoptosis through autophagy-induced. Following treatment with RHSL in the absence or presence of 3-MA (5 mM, **A**) and CQ (100 nM, **D**) for 24 h. Representative immunoblot for quantification of LC3B (**B**,**E**) and cleaved-PARP (**C**,**F**) expression in RIN-m5F. The optical densities of bands in each lane were normalized to GAPDH (loading control) optical density from the same gel. * *p* < 0.05; ** *p* < 0.001 compare with each control group (citrate acid).

To further explore the role of RHSL-induced autophagy on RIN-m5F cells, we used two autophagy inhibitors 3-methyladenine (3-MA) and chloroquine (CQ) to block the formation of autophagosomes. The results showed that treatment with 3-MA inhibited the conversion of LC3-I to LC3-II (Figure 5A,B) and increased the protein level of cleaved-PARP (Figure 5A,C) induced by RHSL. Moreover, CQ treatment facilitated the accumulation of LC3-II since the fusion of autophagosomes and lysosomes is blocked (Figure 5D,E), while increased the expression of cleaved-PARP (Figure 5D,F) induced by RHSL. Taken together, these results showed that RHSL exerts its protective role by activating autophagy and consequently inhibited STZ-induced apoptosis in RIN-m5F cells.

## 4. Discussion

**Figure 6:**
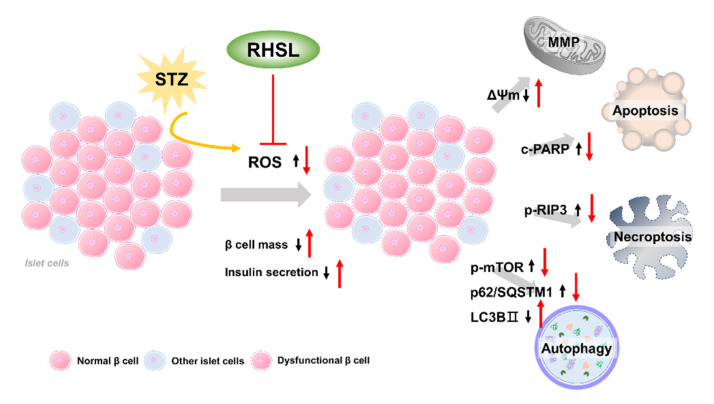
Schematic model of the potential mechanisms of RHSL protective effect on RIN-m5F cells from STZ-induced oxidative damage. Black arrows are the effects of STZ, red arrows are effects of RHSL. Abbreviation: RHSL, rice husk silica liquid; STZ, streptozotocin; ROS, reactive oxygen species; ΔΨm, mitochondria membrane potential; RIP3, receptor-interacting protein 3; PARP, poly (ADP-ribose) polymerase; mTOR, mammalian target of rapamycin; p62/SQSTM1, sequestosome-1; LC3, light chain 3.

The current study provides new insights regarding the potential utility of rice husk silica liquid (RHSL) in T2DM, RHSL abrogated the effect of streptozotocin (STZ) on the mass of RIN-m5F pancreatic β cells and the ability of insulin secretion, via reducing the reactive oxygen species (ROS) production, recovering the depolarization of mitochondrial membrane potential (ΔΨm), as well as reversing the expression of cleaved-PARP, p-RIP3, p-mTOR, p62/SQSTM1, and LC3B protein. The proposed mechanisms of RHSL action in the prevention of STZ-induced RIN-m5F cell death were summarized in Figure 6.

The major pathways associated with STZ-induced pancreatic β cells death include DNA methylation and activation of DNA repair mechanisms [15], nitric oxide (NO) overproduction [16], and glucose auto-oxidation which in turn generates free radicals [15]. Moreover, STZ induces pancreatic β cell toxicity through mitochondrial dysfunction, ROS production, necrosis, and apoptosis [4].

RIN-m5F cells are a widely used insulin-secreting cell line, mainly containing insulin and a small amount of glucagon and somatostatin [17]. STZ administration leads to the rapid destruction of pancreatic β cells, thus massive insulin was released from ruptured β cells to blood [4]. A previous study revealed that a considerable decrease in insulin secretion ability in STZ-treated INS-1 cell, whereas paeoniflorin (PF) pretreatment was capable of improving the insulin secretion ability of INS-1 cells [18]. In addition, 1,25(OH)_2_D3 enhances insulin secretion in STZ-treated d MIN6 cells at low and high glucose concentrations [19]. An in vivo experiment showed that, compared with the control group of diabetic mice, the plasma insulin of the Si treatment group was significantly reversed to near-normal levels [11]. Similar to the results of previous studies, the insulin levels of pancreatic β cells in the STZ treatment group were significantly reduced in our study, while RHSL significantly reversed the insulin level that was reduced by STZ.

Higher levels of ROS are found in the islets of T2DM patients, and their presence attenuates insulin secretion [4]. STZ that induces diabetes is also closely related to the production of ROS. Several studies have demonstrated that STZ induces damage predominantly by provoking the production of ROS and NO in islet cells [4]. In STZ-treated MIN6 β-cells, ROS generation was statistically significantly increased by 55.1 ± 4.7% (*p* < 0.001) [20]. Similarly, AI-Nahdi et al. observed a significant increase in intracellular ROS production in RIN-m5F cells treated with 1 mM STZ for 48 h [21]. Comparably, our study showed that ROS production was increased in RIN-m5F cells following treatment with 2 mM STZ for 24 h, while RHSL slightly reversed the effects caused by STZ. Therefore, we speculated that RHSL can reverse the cytotoxicity and damage induced by STZ by reducing the content of ROS in RIN-m5F cells.

Under the stimulation of oxidative stress, the mitochondrial membrane potential (ΔΨm) will be changed, which is characterized by the disordered membrane potential leads to the opening of mitochondrial pores, thus releases cytochrome C into the cytoplasm, which in turn triggers the downstream apoptotic cascade [22,23]. AI-Nahdi et al. observed a significant loss in the membrane potential after treatment with 10 mM STZ for 24 h [24]. Similarly, our results showed a significant mitochondrial membrane depolarization after treatment with STZ in RIN-m5F cells. Importantly, RHSL treatment showed a significant recovery of the membrane potential in STZ treated cells.

Most studies have shown that pancreatic β cell apoptosis is a key process in the pathogenesis of T1DM and T2DM, so it is important to alleviate or suppress the effect of STZ that induces diabetes. Fucoidan, a brown algae extract, which is used to be explored against diabetic nephropathy, has also been found to reduce the number of apoptotic cells in STZ-treated NIT-1 cell [25]. In addition, Lianga et al. showed that Fudan-Yueyang G. lucidum alleviated the apoptosis in STZ-treated INS-1 cell [26]. In our study, STZ induced the expression of apoptosis-related protein PARP, while RHSL reversed these results and significantly reduced the expression of the cleaved-PARP protein. The contradictory research revealed that the ability of nano-SiO2 to induce ROS and promote apoptosis in normal human hepatic L-02 cells [27], while no significant alteration of ROS levels and cell viability were seen in 48 and 86 nm of SiO2 exposed groups. Therefore, we believe that the size of silicon particles and cell viability are worthy of further research.

Necroptosis is considered to be an alternate cell death mechanism triggered when apoptosis is blocked. Besides the inhibition of caspase-8 prevents apoptosis induction [28], receptor-interacting protein 3 (RIP3) plays a critical role in the switch between apoptotic and necroptotic cell death triggered by tumor necrosis factor (TNF) [29]. In general, RIP1 mediated apoptosis does not require the presence of RIP3, while RIP1 is accompanied by high levels of RIP3 that promote the formation of necrosome complexes and switch cell apoptosis into necroptosis [28]. More specifically, RIP3 increases TNF-induced ROS production via activation of glutamate-ammonia ligase (GLUL), glutamate dehydrogenase 1 (GLUD1), and eventually induce necroptosis [29,30]. At present, there is no literature to investigate the level of RIP1/RIP3 for pancreatic β cells following induction by STZ, while a previous study indicated that STZ-induced renal injury tissue had significantly higher expression levels of RIP1 and RIP3 [31].

Autophagy is a catabolic process of the lysosomal degradation pathway under conditions of stress, which maintains metabolic turnover and homeostasis in pancreatic β cells [32]. The autophagy process involves the inhibition of the mammalian target of rapamycin complex 1 (mTORC1) and the deacetylation of autophagy components, which promotes the fusion and degradation of autophagosomes containing substances to be degraded and lysosomes containing acid hydrolases [32]. Once autophagy is dysregulated, it will also promote the loss of β cell mass and function [33]. Previous research confirms this argument, they found that autophagy-related (ATG) 7 knockout mice showed decreased β-cell mass through increased apoptosis, which ultimately lead to decreased insulin secretion and impaired glucose tolerance [34]. The most representative indicators of autophagic flux include light chain (LC)3 (autophagosome marker) and p62/SQSTM1 (autophagy adaptor), the latter being the link between LC3 and the ubiquitinated substrates [14]. In our study, we found a significant increase in the transition of LC3-I to LC3-II, while p62/SQSTM1 was decreasing after RHSL treatment in STZ-induced RIN-m5F cells, which indicate that RHSL is indeed involved in the process of autophagy regulation.

On the other hand, 3-methyladenine (3-MA) and chloroquine (CQ) are frequently used autophagy inhibitors that function in early autophagy through PI3K inhibition [35] and in late autophagy by blocking the fusion of autophagosomes and lysosomes [36], respectively. In our study, we found that RHSL significantly induced the expression of cleaved-PARP in 3-MA-treated RIN-m5F cells, and similar results were also observed in CQ-treated RIN-m5F cells, these results reveal that RHSL protects RIN-m5F cells by inducing autophagy. Similar results have also been observed in previous studies, vitamin D enhanced autophagy while inhibited apoptosis in STZ-treated MIN6 β-cells [19]. In addition, T. stricta extract was able to significantly reverse the autophagy markers in response to STZ to near normal level in RIN-m5F cells [37].

The factors that determine the bioavailability of Si element include concentration, type of food, and species in which silicon is present [38,39]. Si in serum and tissues exists in free form and can be absorbed and eliminated in urine quickly due to free diffusion across cell membranes [11]. This implies the advantage of long-term intake without excessive retention and accumulation in the body.

## 5. Conclusions

To our knowledge, this is the first study to report the beneficial effect of RHSL on pancreatic β cells. Our results showed that RHSL reversed the ROS production and mitochondria depolarization, and also alleviated STZ-induced apoptosis by inducing autophagy in RIN-m5F cells. This study highlighted the importance of RHSL on supporting the β-cells function, and autophagy has also become an important mechanism for RHSL to improve diabetes in the future.

## Data Availability

The data presented in this study are available on request from the corresponding author. The data are not publicly available due to patent application.
